# Distinct roles of inotropic and metabotropic glutamate receptors in rhythmic activity generated by pre-Botzinger complex

**DOI:** 10.1186/1471-2202-14-S1-P392

**Published:** 2013-07-08

**Authors:** Lee Davis, Natalia Toporikova

**Affiliations:** 1 Biology Department, Washington and Lee University, Lexington, VA, 24450, USA; 2 Computer Science Department, Washington and Lee University, Lexington, VA, 24450, USA

## 

To keep up with oxygen demands, the central nervous system regulates frequency of respiratory rhythm. The inspiratory phase of the respiratory rhythm originates in the pre-Bötzinger complex (preBötC), a region of the ventrolateral medulla. preBötC neurons generate stable rhythmic output when isolated from the rest of respiratory column. The frequency of the respiratory rhythm is modulated via release of excitatory neuromodulators by the Raphe nucleus. The respiratory rhythm observed in the preBötC depends on excitatory neurotransmission between neurons in the population, since blocking excitatory neurotransmission eliminates a coordinated rhythm at the level of the preBötC.

**Figure 1:**
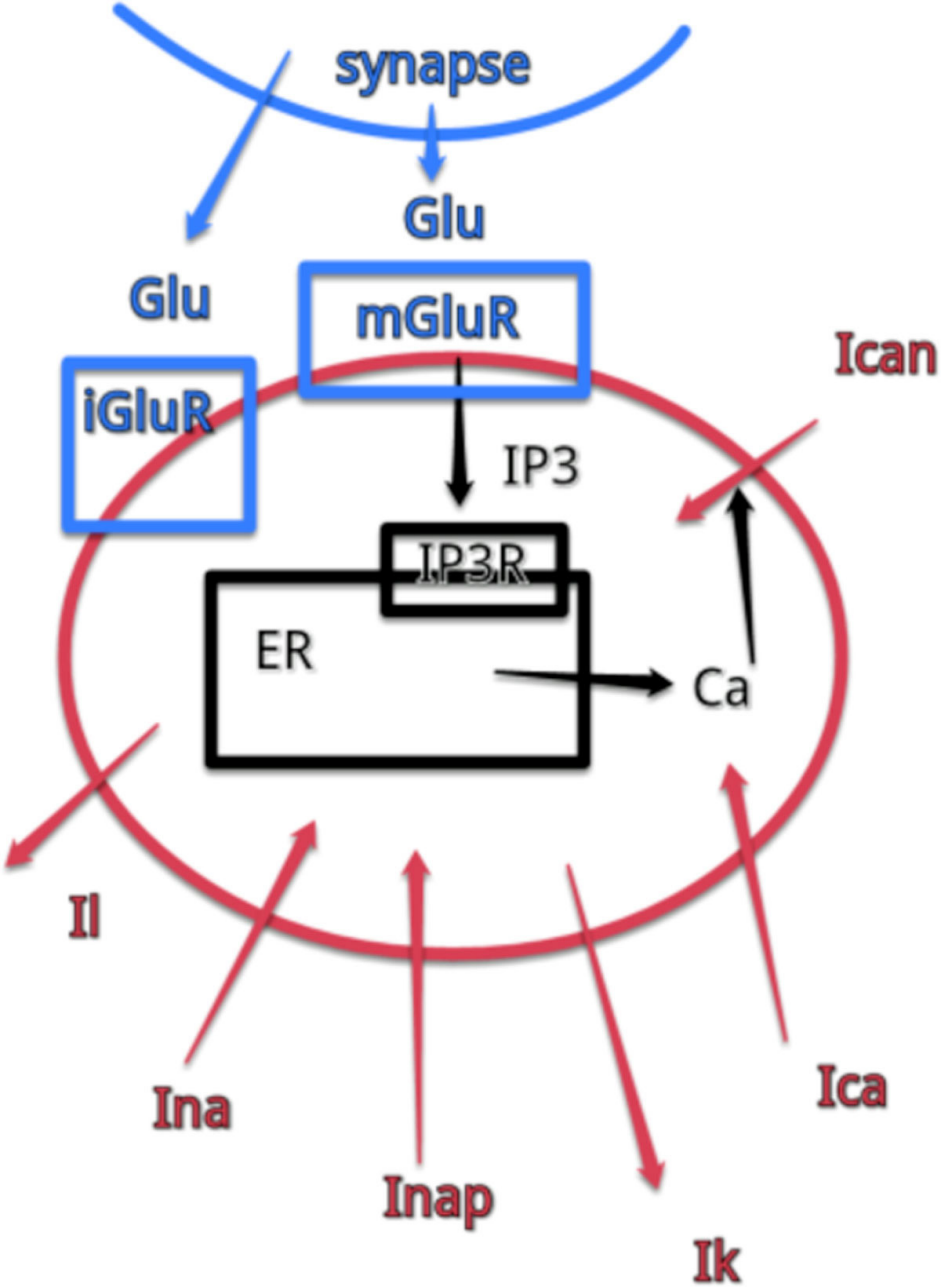
Schematic representation of electrical activity in single preBötC neuron

In this work we extend previously developed model of pre-BotC neuron (Figure 1) to determine the role of ionotropic (iGluR) and metabotropic (mGluR) glutamate receptors on generation of the respiratory rhythm at different levels of neuromodulatory tone. Using distribution of persistent sodium and voltage-gated calcium conductances, we generated the heterogeneous population of neurons connected through both, ionotropic and metabotropic glutamate receptors. Our simulations demonstrate that mGluR and iGluR has distinct roles, determined by neuormodulatory tone. In low neuromodulatory tone, the rhythmic activity of the preBötC network relies on activation of iGluR. In high neuromodulatory tone, the rhythmic activity of the network relies on activation of second messenger cascade by mGluR.

